# The significance of cytoplasmic antinuclear antibody patterns in autoimmune liver disease

**DOI:** 10.1371/journal.pone.0244950

**Published:** 2021-01-07

**Authors:** Hyun Jin Cha, Jimin Hwang, Lucy Eunju Lee, Younhee Park, Jason Jungsik Song

**Affiliations:** 1 Division of Rheumatology, Department of Internal Medicine, Yonsei University College of Medicine, Seoul, South Korea; 2 Synapse Center, Yonsei University College of Medicine, Seoul, Korea; 3 Department of Epidemiology, Johns Hopkins Bloomberg School of Public Health, Baltimore, Maryland, United States of America; 4 Department of Laboratory Medicine, Yonsei University College of Medicine, Seoul, South Korea; 5 Institute for Immunology and Immunological Diseases, Yonsei University College of Medicine, Seoul, South Korea; Texas A&M University, UNITED STATES

## Abstract

We aimed to determine the significance of cytoplasmic antinuclear antibody (ANA) patterns using computer-aided immunofluorescence microscopy in patients with autoimmune liver diseases (AILD). ANA staining pattern was identified by treating cultured human epithelial type 2 (HEp-2) cells with the sera of the patients. Medical records of patients with suspected AILD who had positive cytoplasmic ANA patterns between February 2017 and November 2019 were retrospectively reviewed for clinical, laboratory, and immunological data. Cytoplasmic ANA patterns of AILD and non-AILD groups were compared. Further subgroup analysis of patients with AILD who had reticular or speckled cytoplasmic ANA patterns was conducted. We found that among the 196 patients with positive cytoplasmic ANA patterns, 113 (57.6%) were diagnosed with AILD. The percentage of reticular cytoplasmic pattern was higher in the AILD group than that in the non-AILD group (64.0% vs. 21.9%, p < 0.001). Furthermore, patients with AILD who exhibited a reticular ANA pattern demonstrated a higher positive rate for anti-mitochondrial antibodies (66.7% vs. 2.6%, p < 0.001) than those who exhibited the speckled ANA pattern. Moreover, AILD patients with the reticular ANA pattern displayed a lower positive rate for anti-smooth muscle antibodies (0% vs. 45%, p < 0.001) and nuclear ANA pattern (73.2% vs. 97.5%, p = 0.003) than those with the speckled ANA pattern. Therefore, cytoplasmic ANA patterns could be used to guide AILD characterization in suspected AILD cases, especially as the reticular ANA pattern is strongly associated with AILD. Thus, it is important to check cytoplasmic ANA patterns for AILD evaluation, even when nuclear ANA patterns are negative.

## Introduction

Autoimmune liver disease (AILD) is a rare immune-mediated chronic liver disease with heterogeneous clinical characteristics. AILD includes three major disease entities, autoimmune hepatitis (AIH), primary biliary cholangitis (PBC), and primary sclerosing cholangitis (PSC) [1]. AIH is characterized by liver inflammation of unknown origin, presence of antinuclear antibody (ANA) and anti-smooth muscle antibody (SMA), increased IgG levels, and response to corticosteroids [2]. PBC is characterized by destructive lymphocytic cholangitis and the presence of anti-mitochondrial antibody (AMA) [3]. PSC is an autoimmune cholestatic liver disorder that exhibits a characteristic beaded appearance of the intra- and extra-hepatic bile ducts and is strongly associated with inflammatory bowel disease and cholangiocarcinoma [4]. Some patients exhibit characteristics of both AIH and PBC, thereby comprising a distinct population with the AIH-PBC overlap syndrome [5].

Autoimmune serology has been extensively utilized in AILD diagnosis and characterization. In particular, the ANA test has been traditionally used as a screening test, followed by secondary tests that identify the presence of specific autoantibodies [6]. ANA has been reported to occur in 50–70% of AIH patients [7]. In PBC, the positivity rate of ANA is about 50%, whereas that of AMA is 90%, thus showing specificity [8]. ANA prevalence in PSC varies widely from 8% to 77% [9].

In the clinical setting, the ANA test is typically performed through indirect immunofluorescence assays on human epithelial type 2 (HEp-2) cells, following treatment with the sera from the patients. To mitigate inter-physician heterogeneity in the naming and classification of ANA staining patterns and to reach an international consensus, the 2015 International Consensus on ANA Patterns (ICAP) workshop classified ANA patterns into three major groups, nuclear, cytoplasmic, and mitotic patterns. Nuclear patterns include the distinct staining pattern of the nucleoplasm—speckled or homogeneous—and patterns attributed to specific nuclear subcomponents, i.e., centromere, nuclear dots, nucleolar, or nuclear envelope. Cytoplasmic patterns represent staining of the cytoplasm and are subdivided into five different patterns, i.e., fibrillar, speckled, reticular/mitochondrial, polar/Golgi-like, and rods and rings [10]. These ANA patterns may provide clinically relevant insights into AILD, such as the suspected disease entity and further recommended diagnostic measures [8, 11–13]. Although ANA positivity plays an important role in AILD diagnosis, the clinical implications of cytoplasmic ANA patterns in AILD have not been well defined.

The recent advent of automated, computer-aided immunofluorescence microscopy (CAIFM) has enabled substantial accumulation of ANA staining data and reassessment of previous findings. In addition to enabling more valid and reliable pattern classification, CAIFM allows antibody profiling based on large-scale data on HEp-2 patterns such that ANA patterns can be employed in clinical decision-making [14]. Therefore, in this study, we aimed to utilize stored CAIFM ANA data of patients with suspected AILD to determine the significance of cytoplasmic ANA patterns in AILD.

## Materials and methods

### Patient selection

**Fig 1:**
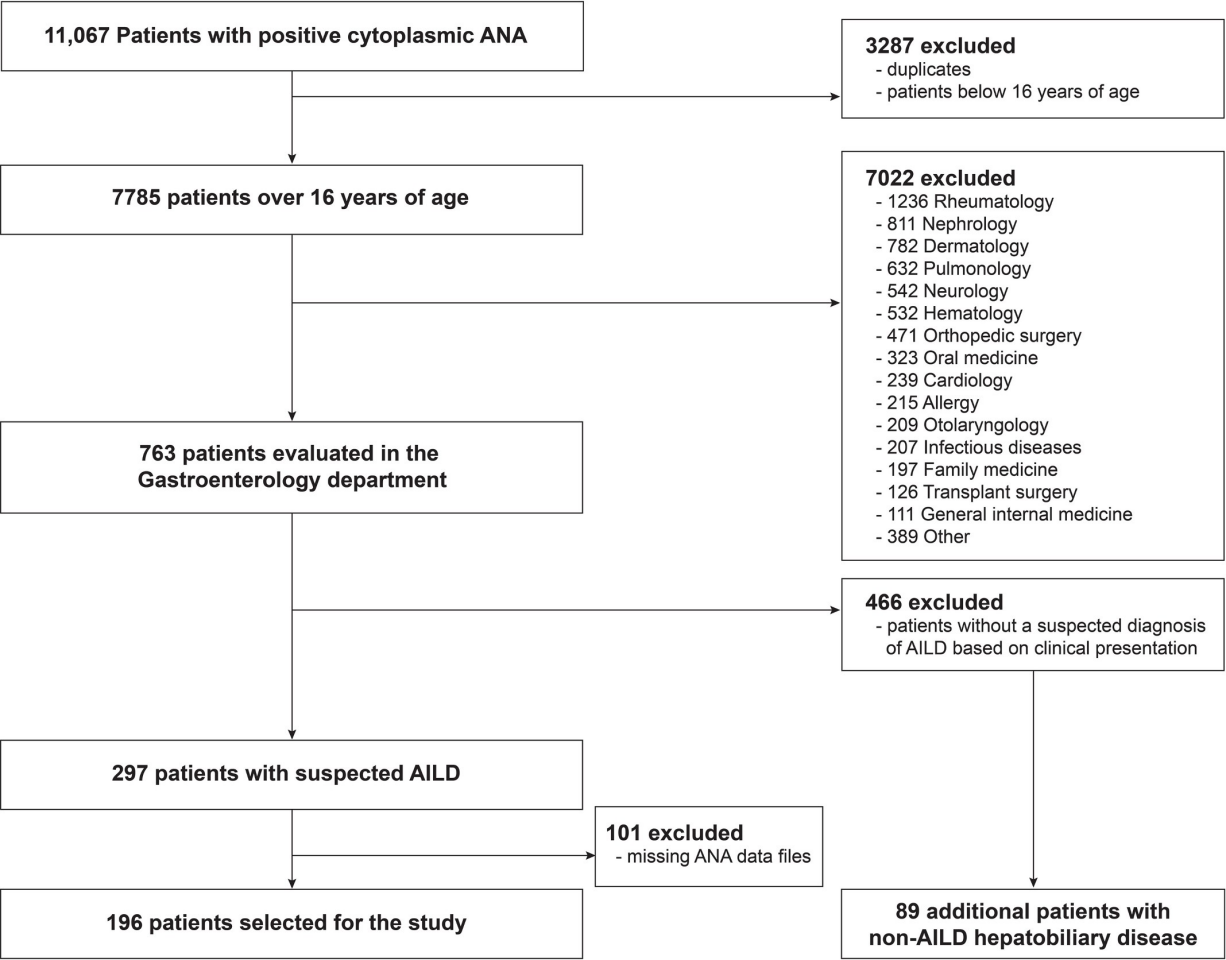
Flowchart for patient inclusion. ANA, antinuclear antibody; AILD, autoimmune liver diseases.

We retrospectively reviewed the medical records of patients who were positive for cytoplasmic ANA staining from the database hosted by the Department of Laboratory Medicine, Severance Hospital, Seoul, Korea, between February 2017 and November 2019. At the time of testing, cytoplasmic ANA was evaluated by indirect immunofluorescence assays on HEp-2 cells, following treatment with the sera from the patients. We screened 11,067 patients with positive cytoplasmic ANA and excluded duplicates and patients below 16 years of age. Of the remaining 7785 patients, we selected 763 patients who were evaluated in the Gastroenterology department. Among them, we then reviewed 297 patients with suspected AILD and further excluded 101 patients whose ANA image data files were missing. Finally, 196 patients with cytoplasmic ANA staining were included in this study (Fig 1). Additionally, we examined the diagnoses of the 466 excluded patients evaluated in the Gastroenterology department and selected an additional 89 non-AILD patients with hepatobiliary diseases for comparison of cytoplasmic ANA patterns. This study was approved by the Institutional Review Board of Severance Hospital and conducted in accordance with the Declaration of Helsinki (IRB approval number: 4-2020-0784). Considering the retrospective study design, the requirement of informed consent was waived.

### Acquisition of clinical and laboratory data

Clinical data including age, sex, and diagnosis were collected. Laboratory data including white blood cell (WBC) count, alkaline phosphatase (ALP), aspartate aminotransferase (AST), alanine aminotransferase (ALT), γ-glutamyl transferase (GGT), serum albumin, and total bilirubin levels obtained within a week from the date of ANA testing were analyzed in this study. Additionally, treatment status was examined by reviewing electronic medical records. Medications that the patient had been receiving within 1 year from the date of the ANA test were noted.

### Immunological profile

Immunological tests included the determination of the presence of the following autoantibodies: ANA, AMA, and SMA. ANA immunofluorescence assays were performed on cultured HEp-2 cells exposed to patient sera using NOVA Lite DAPI ANA Kit (Inova Diagnostics, San Diego, CA, USA) according to the manufacturer's specifications. Briefly, the sera of the patients were diluted 1:80 for screening. The substrate slides with HEp-2 cells were treated with diluted patient serum samples for 30 min. After the slides were washed with phosphate-buffered saline (PBS), they were incubated with fluorescein-labeled anti-human IgG conjugate and DAPI (4′-6-diamidino-2-phenylindol) for 30 min. The slides were washed again with PBS and then covered with coverslips. The slides were evaluated using NOVA View software (version 3.6.0.1), and the results were manually reviewed by two experts. The NOVA View software could recognize and identify five major nuclear patterns (homogenous, nucleolar, centromere, nuclear dot, and speckled) and the presence or absence of cytoplasmic patterns. All image files were stored and could be reviewed at any time. In this study, the stored ANA image files that exhibited cytoplasmic patterns in suspected AILD patients were recalled and manually classified into five major cytoplasmic patterns (fibrillar, speckled, reticular, polar/Golgi-like, and rods and rings). AMA and SMA were tested using the indirect immunofluorescence assay (NOVA Lite ANA KSL Kit, Inova Diagnostics). The screening dilution for AMA and SMA was 1:20. All test slides for AMA and SMA tests were manually analyzed by a laboratory medicine specialist using a fluorescence microscope.

### Definition of cases

According to the simplified scoring system proposed by the International Autoimmune Hepatitis Group (IAIHG), AIH was diagnosed when the pre-treatment simplified score was > 6 and the clinical presentation was consistent with that of AIH [15]. PBC was diagnosed when at least two of the following criteria were met: (1) biochemical evidence of cholestasis with ALP elevation, (2) presence of AMA, and (3) compatible liver histopathology in accordance with the 2009 guidelines of the American Association of the Study of Liver Diseases (AASLD) [16]. Furthermore, patients were diagnosed with AIH-PBC overlap syndrome when both the clinical history and liver biopsy results were typical of the overlap syndrome [17, 18]. Patients were diagnosed with PSC when the following criteria were met: (1) cholestatic biochemical profile, (2) compatible cholangiography or liver histopathology, and (3) absence of secondary causes of cholangitis such as IgG4 disease [19].

### Statistical analysis

Continuous variables were presented as the medians with interquartile ranges (IQRs), whereas categorical variables were expressed as frequencies and percentages. Continuous variables were compared using the Mann-Whitney U test, whereas categorical variables were compared using the Chi-square test or Fisher’s exact test, as appropriate. In all statistical analyses, a two-tailed p-value of < 0.05 was considered significant. Statistical analyses were performed using R version 3.6.2 (R Foundation for Statistical Computing, Vienna, Austria).

## Results

### Baseline characteristics of patients

The demographic and clinical characteristics of 196 patients with suspected AILD included in this study are shown in Table 1. The median age was 57.96 years, and 75.5% of the patients were women. Most patients had reticular (51.0%) or speckled (46.4%) cytoplasmic ANA patterns, while very few patients had fibrillar ANA pattern (2.0%). There were no cases of polar/Golgi-like or rods and rings patterns. Interestingly, nearly a quarter of the patients (23.5%) tested negative for nuclear ANA patterns. Among the 196 patients, 113 (57.6%) exhibited a confirmed diagnosis of AILD, and of those, the majority were diagnosed with either AIH (56.6%) or PBC (26.5%). Furthermore, 17 (15.0%) patients presented with overlap syndrome, and the remaining two patients had PSC. Non-AILD cases included drug-induced hepatitis (20.5%), non-alcoholic fatty liver disease (19.3%), infectious hepatitis (13.3%), chronic hepatitis (13.3%), acute hepatitis (10.8%), cancer (7.2%), systemic autoimmune disease (6.0%), liver cirrhosis (2.4%), alcoholic liver disease (2.4%), and Wilson’s disease (1.2%).

**Table 1:** Demographic and clinical characteristics of the enrolled patients with positive cytoplasmic ANA patterns.

Characteristic	All patients (n = 196)
**Age (years)**	57.96 (49.83–67.69)
**Female**	148 (75.5%)
**ANA screening**	
**Nuclear pattern**	
Negative (<1:80)	46 (23.5%)
Positive (≥1:80)	150 (76.5%)
**Cytoplasmic pattern**	
Fibrillar	4 (2.0%)
Speckled	91 (46.4%)
Reticular	100 (51.0%)
Polar/Golgi-like	0 (0.0%)
Rods and rings	0 (0.0%)
Mixed speckled and reticular	1 (0.5%)
**Diagnosis**	
**Autoimmune liver disease**	**113 (100%)**
Autoimmune hepatitis (AIH)	64 (56.6%)
Primary biliary cholangitis (PBC)	30 (26.5%)
Overlap syndrome (AIH-PBC)	17 (15.0%)
Primary sclerosing cholangitis (PSC)	2 (1.8%)
**Non-autoimmune liver disease**	**83 (100%)**
Drug-induced hepatitis	17 (20.5%)
Non-alcoholic fatty liver disease	16 (19.3%)
Infectious hepatitis^a^	11 (13.3%)
Chronic hepatitis	11 (13.3%)
Acute hepatitis	9 (10.8%)
Cancer^b^	6 (7.2%)
Systemic autoimmune disease	5 (6.0%)
Acute cholangitis	3 (3.6%)
Liver cirrhosis	2 (2.4%)
Alcoholic liver disease	2 (2.4%)
Wilson’s disease	1 (1.2%)

Data are presented as the median (interquartile range) or n (%). ANA, antinuclear antibody

^a^Infectious hepatitis included hepatitis A (n = 2), hepatitis B (n = 5), hepatitis C (n = 1), Epstein-Barr viral hepatitis (n = 5), and hepatitis due to *Clonorchis sinensis* (n = 1).

^b^Cancer included cholangiocarcinoma (n = 3), hepatocellular carcinoma (n = 2), and intraductal papillary mucinous neoplasm (n = 1).

The treatment status of the patients with AILD within 1 year of the ANA test is presented in S1 Table. Common AILD treatments used in the patients—corticosteroids, azathioprine, cyclosporine, and ursodeoxycholic acid (UDCA)—were tabulated. Of note, 53.1% and 26.5% of the patients with AIH were on corticosteroids and immunosuppressants (azathioprine or cyclosporine), respectively, whereas only 6.7% of the patients with PBC were on corticosteroids. Among patients with AIH-PBC, 47.1% were receiving corticosteroids, and 17.6% were receiving azathioprine. Most patients with AILD (95.6%) were receiving UDCA.

### Cytoplasmic ANA patterns of patients with and without AILD

**Fig 2:**
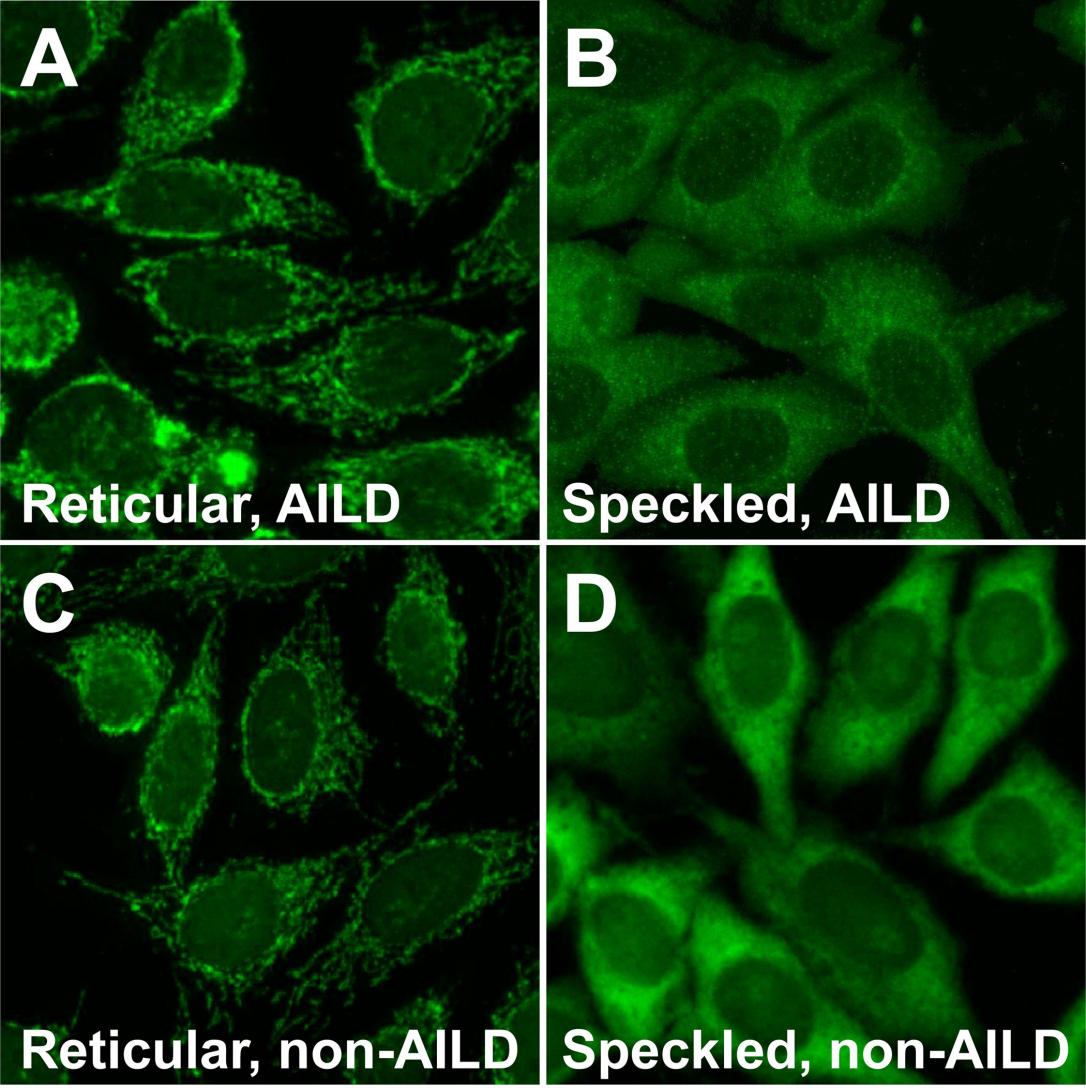
Representative images of the cytoplasmic antinuclear-antibody staining pattern in HEp-2 cells by using the sera of AILD or non-AILD patients. The reticular pattern shows granular and filamentous staining throughout the cytoplasm, whereas the speckled pattern shows fine speckles distributed throughout the cytoplasm. The reticular (A) and speckled (B) patterns in patients with primary biliary cholangitis, the reticular pattern in a patient with hepatitis B (C), and the speckled pattern in an osteoarthritis patient (D).

We compared the cytoplasmic ANA staining patterns of patients with and without AILD. In the analysis, we also included 89 non-AILD hepatobiliary patients. The representative images for the speckled and reticular cytoplasmic ANA staining patterns are shown (Fig 2). The proportion of the reticular cytoplasmic ANA pattern was higher in the AILD group than that in non-AILD group (64.0% vs. 21.9%, p < 0.001) (Table 2).

**Table 2:** Cytoplasmic ANA patterns of patients with and without AILD.

Cytoplasmic pattern	AILD (n = 111)	non-AILD (n = 169)	p-value
Reticular	71 (64.0%)	37 (21.9%)	< 0.001
Speckled	40 (36.0%)	132 (78.1%)	

Data are presented as n (%); p-values were calculated using Fisher’s exact test. ANA, antinuclear antibody; AILD, autoimmune liver disease

### Cytoplasmic ANA patterns among patients with AILD

We found that among the patients exhibiting the speckled cytoplasmic pattern, a majority (80%) were diagnosed with AIH (Table 3). Patients with the reticular pattern showed similar prevalence of AIH and PBC (45.1% and 38.0%, respectively). Furthermore, patients with the reticular ANA pattern also exhibited higher positivity rate of AMA (66.7% vs. 2.6%, p < 0.001) and lower positivity rate of SMA (0.0% vs. 45.5%, p < 0.001) compared with those with the speckled ANA pattern. A lower proportion of patients exhibited positive nuclear ANA patterns in the reticular group than that in the speckled group (73.2% vs. 97.5%, p = 0.003). In addition, patients in the reticular group showed lower AST (36.0 vs. 110.0 IU/L, p < 0.001), ALT (29.0 vs. 61.0 IU/L, p < 0.001), and total bilirubin (0.80 vs. 1.25 mg/dL, p = 0.002) levels, and higher albumin levels (4.10 vs. 3.70 g/dL, p = 0.005) than patients in the speckled group. Further analyses were conducted to examine the association of cytoplasmic ANA patterns with titers and patterns of nuclear ANA (Table 4). Patients in the reticular group exhibited a higher proportion of nuclear ANA titers (> 1:640) than patients in the speckled group (50.0% vs. 10.8%, p < 0.001). Patients in the reticular group also had a higher proportion of the centromere-type nuclear ANA pattern than patients in the speckled group (20.0% vs. 2.6%, p = 0.031).

**Table 3:** Clinical characteristics of patients with AILD based on the cytoplasmic ANA patterns.

	Cytoplasmic pattern		
Characteristic	Reticular (n = 71)	Speckled (n = 40)	p-value
**Diagnosis**			
AIH	32 (45.1%)	32 (80.0%)	0.002
PBC	27 (38.0%)	3 (7.5%)	
AIH-PBC	11 (15.5%)	4 (10.0%)	
PSC	1 (1.4%)	1 (2.5%)	
**Age (years)**	57.50 (51.12–67.58)	58.92 (50.23–67.77)	0.861
**Female**	60 (84.5%)	33 (82.5%)	0.994
**Autoantibodies**			
AMA positive	42/63 (66.7%)	1/39 (2.6%)	< 0.001
SMA positive	0/40 (0.0%)	10/22 (45.5%)	< 0.001
ANA positive (≥1:80, nuclear)	52/71 (73.2%)	39/40 (97.5%)	0.003
**Laboratory findings**			
WBC count (/μL)^a^	5.75 (4.83–6.44)	5.25 (3.86–6.64)	0.393
AST (IU/L)^b^	36.0 (25.0–54.0)	110.0 (45.0–321.0)	< 0.001
ALT (IU/L)^b^	29.0 (17.0–51.5)	61.0 (31.5–226.5)	< 0.001
ALP (IU/L)^b^	129.0 (90.0–221.0)	138.0 (98.5–177.5)	0.956
GGT (IU/L)^c^	123.0 (57.0–290.5)	159.0 (96.0–272.2)	0.507
Albumin (g/dL)^b^	4.10 (3.90–4.35)	3.70 (3.05–4.25)	0.005
Total bilirubin (mg/dL)^d^	0.80 (0.60–1.10)	1.25 (0.80–4.35)	0.002

Data are presented as the median (interquartile range) or n (%); p-values were calculated using the Mann-Whitney U test, the Chi-square test, or Fisher’s exact test, as appropriate. AILD, autoimmune liver disease; AIH, autoimmune hepatitis; PBC, primary biliary cholangitis; PSC, primary sclerosing cholangitis; ANA, antinuclear antibody; AMA, anti-mitochondrial antibody; SMA, anti-smooth muscle antibody; WBC, white blood cell; AST, aspartate aminotransferase; ALT, alanine aminotransferase; ALP, alkaline phosphatase; GGT, γ-glutamyl transferase

^a^Number confined to patients who underwent each test (n = 95).

^b^Number confined to patients who underwent each test (n = 106).

^c^Number confined to patients who underwent each test (n = 89).

^d^Number confined to patients who underwent each test (n = 105).

**Table 4:** Nuclear patterns and titers of ANA in patients with AILD based on the cytoplasmic ANA patterns.

	Cytoplasmic pattern		
	Reticular (n = 52)	Speckled (n = 39)	p-value
**ANA titers (nuclear)**			
≤1:640	20/40 (50.0%)	33/37 (89.2%)	< 0.001
>1:640	20/40 (50.0%)	4/37 (10.8%)	
**ANA pattern (nuclear)**^a^			
Centromere	10/50 (20.0%)	1/39 (2.6%)	0.031
Homogeneous	13/50 (26.0%)	16/39 (41.0%)	0.203
Speckled	24/50 (48.0%)	29/39 (74.4%)	0.021
Dense fine speckled	1/50 (2.0%)	0/39 (0.0%)	NA^b^
Nuclear dots	8/50 (16.0%)	1/39 (2.6%)	0.083
Nucleolar	1/50 (2.0%)	0/39 (0.0%)	NA^b^

Data are presented as n (%) or n/N (%); p-values were calculated using Fisher’s exact test or the Chi-square test, as appropriate. ANA, antinuclear antibody; AILD, autoimmune liver disease

^a^Number confined to patients who underwent ANA pattern analysis (n = 89).

^b^Not available

### Subgroup analysis of patients with the reticular cytoplasmic ANA pattern

We compared AMA positivity among 63 patients with AILD who exhibited the reticular cytoplasmic ANA pattern (Table 5). Among the 42 patients who were positive for AMA, more than half (54.8%) were diagnosed with PBC, followed by AIH (26.2%) and overlap syndrome (19.0%). Among the 21 patients who were negative for AMA, 14 patients (66.7%) were diagnosed with AIH and only 4 patients (19.0%) were diagnosed with PBC.

**Table 5:** Subgroup analysis of patients with reticular cytoplasmic ANA pattern based on AMA serology.

Diagnosis	Reticular cytoplasmic ANA pattern (n = 63)		p-value
	AMA (+) (n = 42)	AMA (-) (n = 21)	
PBC	23 (54.8%)	4 (19.0%)	0.003
AIH	11 (26.2%)	14 (66.7%)	
AIH-PBC	8 (19.0%)	2 (9.5%)	
PSC	0 (0.0%)	1 (4.8%)	

Data are presented as n (%); p-values were calculated by Fisher’s exact test. ANA, antinuclear antibody; AMA, anti-mitochondrial antibody; AIH, autoimmune hepatitis; PBC, primary biliary cholangitis; PSC, primary sclerosing cholangitis

## Discussion

Nuclear ANA patterns have been utilized to characterize AILD based on its clinical and prognostic relevance [7]. AIH is subclassified into types 1 and 2 (AIH-1 and AIH-2) based on the serological profile; AIH-1 involves the presence of ANA and SMA, while AIH-2 involves the presence of anti-liver kidney microsomal antigen type-1 (LKM1) and anti-liver cytosol type 1 (LC1). Approximately 75% of the AIH-1 patients display a homogeneous pattern, and the remainder display a speckled pattern of nuclear ANA [20, 21]. PBC also is associated with multiple nuclear dots or rim-like membranous nuclear pattern of ANA, which are very specific findings (>95%). These two staining patterns provide strong evidence of PBC, which is especially useful for diagnosing 5–10% of the patients with PBC who are negative for AMA [22, 23]. These staining patterns can be relevant for distinguishing AIH from PBC, because patients with PBC will very rarely present a homogeneous pattern, and patients with AIH alone will not typically display PBC-specific staining patterns [20].

In contrast to the robust literature on nuclear ANA patterns, cytoplasmic staining patterns of ANA have been gaining recognition recently. Cytoplasmic staining may not be reported as routinely as nuclear staining, but it is now becoming accepted as clinically relevant [24, 25]. Specifically, the speckled cytoplasmic pattern, associated with antigens such as tRNA synthetase, ribosomal P proteins, and signal recognition particle (SRP), is observed in systemic lupus erythematosus (SLE), anti-synthetase syndrome, interstitial lung disease, and inflammatory muscle diseases [11]. However, much is yet to be revealed about the prognostic and clinical role of cytoplasmic ANA patterns.

In this study, among the patients clinically suspected with AILD who exhibited a positive cytoplasmic ANA pattern, 57.6% were diagnosed with AILD. Therefore, AILD should be suspected in patients with positive cytoplasmic ANA staining pattern, especially when the clinical presentation aligns with AILD. Of note, the reticular cytoplasmic pattern is a distinct pattern that correlates with the presence of anti-mitochondrial antibodies. Therefore, if a patient with unexplained chronic hepatitis exhibits a reticular cytoplasmic ANA pattern, the physician should suspect AIH or PBC and further test for anti-mitochondrial antibodies. AILD is difficult to diagnose in its early stages, and is only diagnosed through liver biopsy in many cases [26]. It should therefore be useful to check the cytoplasmic pattern of ANA—in addition to nuclear ANA patterns—to establish a diagnosis. Furthermore, in the current study, 26.8% of AILD patients with the reticular cytoplasmic ANA pattern showed negative nuclear ANA pattern. We can thus conclude that having negative nuclear ANA does not preclude the patient from having positive cytoplasmic ANA patterns. Clinicians should consider assessing patients with suspected AILD for positive cytoplasmic patterns, even when they have negative nuclear ANA.

Previous studies have reported that the reticular cytoplasmic ANA pattern is associated with PBC, and in some cases, with systemic sclerosis [11]. In the present study, the reticular ANA pattern was not only highly associated with PBC but was also observed in patients with AIH. Moreover, our study showed that 66.7% of the patients with the reticular cytoplasmic ANA pattern tested positive for AMA; among these patients, 54.8% were diagnosed with PBC. Among patients with AILD who exhibited a speckled cytoplasmic ANA pattern, only 1 (2.6%) patient, diagnosed with AIH, tested positive for AMA. Therefore, testing for AMA is the potential next step for patients exhibiting a reticular cytoplasmic ANA pattern. Interestingly, none of the patients who exhibited a reticular cytoplasmic ANA pattern was positive for SMA. Additionally, our study revealed that the levels of ALT, AST, and bilirubin are significantly lower in AILD patients with reticular cytoplasmic ANA staining than those in patients exhibiting speckled pattern of cytoplasmic ANA staining. Therefore, it is possible that cytoplasmic ANA pattern might be associated with the clinical presentation or prognosis of AILD.

Furthermore, our study showed that centromere-type nuclear ANA pattern was significantly associated with the reticular cytoplasmic ANA pattern. Anti-centromere antibody is an immunological marker of systemic sclerosis, which is known to be associated with the propensity of developing AILD [27, 28]. Herein, among 11 patients with positive anti-centromere nuclear pattern, one patient was diagnosed with systemic sclerosis, whereas the rest did not manifest any clinical signs of systemic sclerosis. Of note, among the remaining ten patients, four patients had AIH, two patients had PBC, and four patients had AIH-PBC overlap syndrome. As only a few reports have suggested an association between the presence of anti-centromere antibodies and AILD, future studies are warranted to investigate the role of anti-centromere antibodies in AILD [29–31].

To the best of our knowledge, the current study is the first to investigate the significance of the reticular cytoplasmic ANA pattern in patients with AILD. By analyzing stored ANA data collected by CAIFM, we ensured the reliability of cytoplasmic ANA patterns. We only included patients with AILD who were diagnosed based on the IAIHG and AASLD guidelines, thereby further validating our results. However, our study has limitations. First, because the study population consisted only of patients who were evaluated for AILD at a single tertiary medical center, our study group cannot be assumed to represent the general population without AILD. Second, our study was cross-sectional, not designed to evaluate the prognosis of AILD, because it requires long term follow-up data. CAIFM is a relatively new technology, and we only started to use CAIFM for ANA testing in 2017 at our hospital. Therefore, it is still early to evaluate long term prognosis. In the future, large-scale, longitudinal studies should be performed to better assess the significance and prognostic value of cytoplasmic ANA patterns in AILD patients, compared with those in the general population. Additionally, though the current study assessed the cytoplasmic ANA patterns by exposing HEp-2 cells to patient sera, further insights could be gained through assessing staining patterns in hepatocytes and cholangiocytes, which are more relevant to AILD. Further studies to elucidate the molecular mechanisms underlying the cytoplasmic reticular pattern mediated by autoantibodies is necessary. In conclusion, cytoplasmic ANA patterns could play a central role in AILD evaluation, and thus, should be taken into account when considering further diagnostic and therapeutic measures for AILD.

## Supporting information

S1 Table Treatment status of patients with AILD according to diagnosis. (DOCX)

S1 Data (XLSX)
