# Supplementary material for: The significance of cytoplasmic antinuclear antibody patterns in autoimmune liver disease
Source: PLoS One. 2021 Jan 7;16(1):e0244950. doi: 10.1371/journal.pone.0244950 (PMC7790257; doi:10.1371/journal.pone.0244950)
Supplement: S1 Table — (DOCX) [file pone.0244950.s001.docx]

**S1 Table. Treatment status of patients with AILD according to diagnosis.**

| **Treatment** | AIH (n=64) | PBC (n=30) | PSC (n=2) | AIH-PBC (n=17) |
| --- | --- | --- | --- | --- |
| Corticosteroids | 34 (53.1%) | 2 (6.7%) | 2 (100.0%) | 8 (47.1%) |
| Azathioprine | 15 (23.4%) | 1 (3.3%) | 0 (0.0%) | 3 (17.6%) |
| Cyclosporine | 2 (3.1%) | 0 (0.0%) | 0 (0.0%) | 0 (0.0%) |
| UDCA | 61 (95.3%) | 29 (96.7%) | 2 (100.0%) | 16 (94.1%) |
